# Chloroquine Enhances Chemosensitivity of Breast Cancer via mTOR Inhibition

**DOI:** 10.3390/biomedicines13040948

**Published:** 2025-04-12

**Authors:** Zhihao Lin, Yuting Xu, Mifang Li, Yibiao Liu, Jianbo Yu, Lingyan Zhang

**Affiliations:** 1 Shenzhen Clinical Medical College, Guangzhou University of Chinese Medicine, Shenzhen 518116, China; 20221111591@stu.gzucm.edu.cn (Z.L.); 20221111594@stu.gzucm.edu.cn (Y.X.); 2 Longgang Central Hospital of Shenzhen, Shenzhen 518116, China; 15626430473@163.com (M.L.); liuyibiao12345@126.com (Y.L.); 3 Lab of Molecular Imaging and Medical Intelligence, Department of Radiology, Longgang Central Hospital of Shenzhen, Shenzhen 518116, China; 4 Longgang Clinical Institute of Shantou University Medical College, Shenzhen 518116, China

**Keywords:** chloroquine, mTOR, P2X4, chemotherapy, triple-negative breast cancer

## Abstract

**Background**: Chloroquine (CQ) has been extensively validated for its safety as an antimalarial drug. The treatment regimen combining CQ with 5-fluorouracil (5-FU) has demonstrated promising antitumor effects in both in vitro and animal models. However, the clinical application of this combination therapy still faces numerous challenges, primarily due to the unelucidated mechanistic underpinnings. **Methods**: We validated the synergistic effect of CQ in antitumor therapy using 5-fluorouracil and N-acetylcysteine. Subsequently, we employed lysosomal pH probes and inhibitors (5-BDBD and bafilomycin A1) to verify the mechanism of CQ in synergistic antitumor therapy. Finally, the therapeutic efficacy and underlying mechanisms of CQ were further confirmed through in vivo experiments. **Results**: Here, we found that CQ can inhibit the ATP-induced activation of mammalian target of rapamycin (mTOR), enhancing the inhibition of 5-FU on the proliferation and survival of tumors. Mechanistically, CQ affects the lysosomal pH value, leading to the inhibition of P2X4 receptor activity. The ATP-P2X4-mTOR axis is consequently disrupted, resulting in the weakened activation of mTOR. **Conclusions**: Our findings suggest that CQ may inhibit ATP-induced mTOR activation by suppressing P2X4 receptor signaling, thereby altering the apoptosis resistance of tumors. The combination of CQ and 5-FU represents a promising therapeutic strategy, particularly for mTOR-hyperactivated malignancies refractory to conventional chemotherapy. These findings not only advance our understanding of the mechanisms underlying CQ-based combination therapy but also highlight the therapeutic potential of pharmacologically targeting mTOR and its alternative pathways in combination chemotherapy regimens.

## 1. Introduction

Chloroquine (CQ), a well-established antimalarial agent with an excellent safety profile, has garnered significant preclinical validation as a potential adjuvant in cancer therapy [1,2]. Its chemosensitizing properties stem from the ability to potentiate tumor responses to various chemotherapeutic agents through multifaceted mechanisms [3,4]. While numerous clinical trials have focused on CQ’s role as an autophagy flux inhibitor [5], the heterogeneity of trial designs—encompassing diverse tumor types, disease stages, and combination regimens—has contributed to inconsistent clinical outcomes [6]. Beyond autophagy modulation, emerging evidence reveals that CQ directly targets key metabolic enzymes: choline kinase alpha (CHKA) in phospholipid metabolism and ATP-dependent 6-phosphofructokinase muscle type (PFKM) in glycolysis. This dual enzymatic inhibition disrupts PI3K/Akt/mTOR signaling and counteracts the Warburg effect, two hallmarks of cancer cell survival [7]. Furthermore, CQ functions as an AMPK agonist [8], mirroring the therapeutic synergy observed when combining AMPK activators (AICAR) with mTOR inhibitors to achieve dose-sparing effects [9]. Notably, CQ suppresses amino acid-driven mTOR activation through lysosomal pH modulation, establishing a direct mechanistic link between its metabolic reprogramming capacity and mTOR pathway regulation [10]. However, the precise interplay between CQ-mediated mTOR regulation and its chemosensitization efficacy remains incompletely defined, particularly regarding tumor type-specific pathway dependencies. This knowledge gap underscores the need for predictive biomarkers to optimize CQ combination strategies in precision oncology.

Mammalian target of rapamycin (mTOR) is a critical cellular signaling protein [11,12], and the mTOR pathway has been found to be overactivated in various types of cancers [13], promoting their growth and proliferation. For instance, mTOR signaling is often excessively activated in hepatocellular carcinoma, and its inhibition, in combination with cell division cycle 7 suppression, can effectively induce apoptosis in liver cancer cells [14]. This indicates that mTOR is a valuable therapeutic target for tumors. Furthermore, the combination of mTOR inhibitors with chemotherapy and photodynamic therapy has shown significant efficacy in the treatment of multiple tumors [15,16]. However, the development of mTOR inhibitors is hampered by issues such as adverse reactions and insufficient targeting [17,18,19]. Therefore, identifying alternative targets for the inhibition of the mTOR pathway is an ideal approach. Here, we demonstrate the inhibitory effect of CQ on the ATP-induced mTOR pathway in 4T1 cells, revealing that the sensitizing effect of CQ on 5-fluorouracil (5-FU) partially depends on the mTOR pathway.

## 2. Materials and Methods

1. Materials

Chloroquine, bafilomycin A1, dexamethasone (DEX), and 2′,7′-Dichlorodihydrofluorescein diacetate (DCFH-DA) were purchased from MCE (Monmouth Junction, NJ, USA). 5-BDBD and rapamysin were purchased from topscience (Shanghai, China). Lysosensor yellow/blue-DND 160 (PDMPO) and lysosensor green DND-189 were purchased from yeasen (Shanghai, China). The Click-iT EdU-488 cell proliferation test kit and thiazole blue (MTT) were purchased from Servicebio (Wuhan, China). All other reagents were supplied by beyotime biotechnology Co., Ltd. (Shanghai, China).

2. Cell culture

The mouse breast cancer cell line 4T1 was purchased from the Chinese Academy of Sciences cell bank. The 4T1 cells were cultured in RPMI 1640 (Gibco, Waltham, MA, USA) containing 10% fetal bovine serum (TransSerum^®^ FQ Fetal Bovine Serum FS301, TransGen Biotech, Beijing, China) in a humidified incubator at 37 °C under 5% CO_2_.

3. EDU proliferation assay

The 4T1 cells were plated in a glass-bottom dish and incubated for 24 h. The cells were then treated with a medium containing EdU solution for 2 h, followed by washing with PBS three times. Fixation was performed using a fixation solution composed of 4% paraformaldehyde in PBS at room temperature for 15 min, followed by three washes with PBS, each lasting 5 min. To permeabilize the cells, a solution of 0.3% Triton X-100 in PBS was applied for 15 min at room temperature, followed by three additional washes with PBS, each lasting 5 min. The click reaction mixture was added for 1 h reaction in the dark. DAPI staining solution was then added, and the cells were incubated at room temperature in the dark of 10 min. Images were collected using a fluorescence microscope and analyzed with ImageJ (1.53c).

4. Measurement of ROS and ATP

ROS levels were measured using 2′,7′-Dichlorodihydrofluorescein diacetate (DCFH-DA) (MCE, Monmouth Junction, NJ, USA). Briefly, 4T1 cells were treated as indicated with 5-FU, stained with DCFH-DA (1 µM). Then, the cells were washed with PBS three times and analyzed by fluorescence microscopy.

ATP release was determined with an ATP assay kit. Cells were seeded into a 96-well plate and incubated for 24 h. Then, the transfected cells were incubated with 5-FU as indicated in fresh medium. Then, the cell culture medium was collected for analysis. In total, 100 μL of ATP detection reagent was added to 20 μL of sample or standard solution. The luminescence of the samples was measured by a FlexStation 3 Multi-Mode Microplate Reader (Danaher Corporation, Washington, DC, USA) for ATP determination.

5. Immunoblot analysis

After cell treatment, total protein was extracted using RIPA lysis buffer containing a mixture of protease and phosphatase inhibitors. The samples were then processed with 5x loading buffer and subjected to heat denaturation, followed by separation using 12% SDS-PAGE. The proteins were transferred to a 0.22 μM nitrocellulose membrane, which was sealed with 5% non-fat dry milk for 2 h. The membrane was incubated for 1.5 h at room temperature with a mixture of antibodies against P2X4 receptor (1:1500), phosphorylated ribosomal protein S6 (p-S6) (1:1500), ribosomal protein S6 (S6) (1:1000), GAPDH (1:2000), and β-actin (1:2000). Subsequently, HRP-conjugated goat anti-rabbit IgG (1:2000) and goat anti-mouse IgG (1:2000) were incubated at room temperature for 2 h. Detection was performed using the ECL chemiluminescent reagent.

6. Real-time fluorescent quantitative reverse transcription PCR (RT-qPCR)

Firstly, place the cells in an enzyme free centrifuge tube, add 1 mL of Trizol, and use a super Crush with a sound crusher. Let it stand at room temperature for 10 min, then centrifuge at 12,000 rpm for 5 min, take the supernatant, and follow these steps. Add 100 μL chloroform substitute, invert and mix well, and leave at room temperature for 10 min. Centrifuge at 12,000 rpm, 4 °C, for 15 min, carefully aspirate the upper aqueous phase, transfer it to another centrifuge tube, add an equal volume of isopropanol, mix well, and leave at 4 °C for 10 min. Centrifuge at 12,000 rpm, 4 °C, for 10 min, discard the supernatant, and add 500 μL clean water once with 75% ethanol. Open the lid and air-dry the solution at room temperature, then add 300 μL RNase free water. Dissolve the RNA precipitation in L’s RNase free water and vortex at room temperature for 5 min to fully dissolve the RNA. The reverse transcription solution was prepared as follows: total RNA (1 μg), Enzymes Mix (2 μL), 5× RT SuperMix Buffer (4 μL), and RNase-free ddH_2_O were adjusted to a final volume of 20 μL. After gently pipetting and mixing, the solution was briefly centrifuged. The reaction was incubated at 37 °C for 2 min, followed by incubation at 50 °C for 15 min, and finally heated at 85 °C for 2 min. The mixture was then placed on ice for subsequent experiments. Real time-quantitative PCR was performed using 2X M5HiPer SYBR Premix Es Taq and CFX CONNECT. 18s RNA primers as controls.

7. Colocalization analysis of P2X4 and LAMP1 immunostaining

For immuno-fluorescence staining, the cells were fixed in methanol for 15 min at −20 °C. After blocking with 10% BSA in PBS, the cells were incubated with P2X4 (Proteintech, 1:200, Wuhan, China) and Lamp1 (Beyotime, 1:100, Shanghai, China) in 2% BSA in PBS containing 0.1% Tween-20 overnight at 4 °C. After washing and staining with secondary antibody for 2 h at room temperature, the merged figures were analyzed by ImageJ (1.53c).

8. Lysosomal pH measurement

Changes in lysosomal pH were detected using the lysosensor yellow/blue DND-160 (PDMPO) and lysosensor green DND-189. After cell treatment, 4T1 cells were treated with 1 μM lysosensor for 10 min at 37 °C. The cells were then washed twice. The fluorescence intensity of lysosensor yellow/blue DND-160 was measured at Ex-360/Em-440 and Ex-360/Em-550. The lysosensor green DND-189 fluorescence intensity was measured at Ex-440/Em-510.

9. MTT assay

A tetrazolium salt (MTT) assay was performed to evaluate cell proliferation/inhibition rate. Briefly, 4T1 cells were cultured for 24 h, and cell viability was measured after incubation with MTT (0.5 mg mL^−1^) during 2 h at 37 °C. The resulting formazan crystals were dissolved in DMSO, and the absorbance was measured at 490 nm using a plate reader.

10. Intracellular calcium concentration assay

The 4T1 cells were increased with 1 μm Fluo-4 AM in PBS. Calcium concentration was determined by the Fluo-4 AM mean fluorescence intensity with ATP stimulated. The fluorescence signal of Fluo-4 AM was detected by a plate reader.

11. Treatment in vivo

BALB/c mice (female, 18.0–20.0 g, 5–6 weeks) were used in an in vivo study. All animal experiments and research procedures were in accordance with the corresponding national standards [20]. The 4T1 tumor-bearing mice model was established by a subcutaneous injection of 4T1 cells (5 × 10^6^ per mouse) into the right armpit region. The tumor volume (V) was calculated by the following equation:

$$V=\frac{length\times {width}^{2}}{2}$$

The mice were randomly divided into five groups (*n* = 6). NAC (0.5%) was dissolved in drinking water and applied 3 days before starting the treatment with 5-FU and CQ. 5-FU (20 mg kg^−1^) and CQ (50 mg kg^−1^) were dissolved in sterile H_2_O and injected intraperitoneally for the times indicated in individual experiments. The therapeutic effect of each group was monitored by measuring the tumor volumes. Moreover, the body weight of the 4T1 tumor-bearing mice was also collected to assess the systemic toxicity of virus complexes. The mice were sacrificed on day 7 and tumors were excised, fixed in 4% paraformaldehyde, and paraffin-embedded. The histological slices were stained with hematoxylin and eosin (H&E) and Ki67.

12. Statistical analysis

All quantitative data were expressed as mean ± standard deviation. The normality and homogeneity of variance assumptions were verified using the Shapiro–Wilk test and Levene’s test, respectively. Statistical analyses were performed using GraphPad Prism software (version 9.0) with appropriate multiple comparison corrections (Benjamini–Hochberg procedure). The sample size for in vivo experiments was predetermined by power analysis (α = 0.05; power = 0.8) using G*Power software (3.1.9.7). Statistical significance was determined by a two-tailed Student’s *t*-test or one-way ANOVA followed by post hoc tests, with * *p* < 0.05, ** *p* < 0.01, *** *p* < 0.001, and **** *p* < 0.0001 denoting significance levels.

## 3. Results

### 3.1. CQ-Mediated Chemosensitization Prevented by N-Acetylcysteine

**Figure 1:**
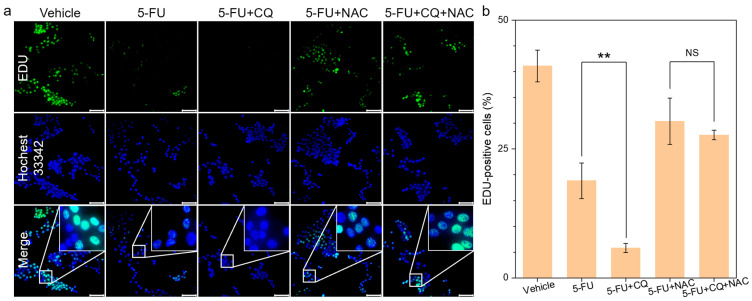
(**a**) EDU fluorescence images of different treatment groups using a fluorescence microscope (5-FU, 5 μM; CQ, 25 μM; NAC, 2 mM) (scale bar, 100 μm). (**b**) Quantitative analysis of the proportion of proliferating cells in different treatment groups (*n* = 3). NS *p* > 0.05, ** *p* < 0.01.

CQ has been reported to enhance the chemotherapeutic effects of 5-FU [21], while N-acetylcysteine (NAC) can alleviate apoptosis by scavenging free radicals and reducing oxidative stress [22]. Therefore, we employed 5-FU and NAC to explore the potential mechanisms underlying the adjuvant chemotherapeutic effects of CQ. We assessed the proliferation activity of 4T1 cells in different treatment groups using the EDU proliferation assay; green fluorescence indicated proliferating cell nuclei, and blue fluorescence represented the total cell count. Analysis through fluorescence imaging (Figure 1a) and quantitative analysis (Figure 1b) revealed a decrease in the proliferation activity of 4T1 cells following 5-FU treatment, with CQ significantly enhancing the inhibitory effects of 5-FU. However, the addition of NAC further mitigated the inhibitory effects of 5-FU, irrespective of whether CQ was included (evidenced by an increased EDU positivity rate). There were no significant differences in tumor proliferation inhibition between the 5-FU + NAC and the 5-FU + CQ + NAC groups. CQ alone did not inhibit 4T1 proliferation (Supplementary Figure S1). These results demonstrate that 5-FU-mediated oxidative stress contributes to apoptosis, and CQ assists in oxidative stress.

### 3.2. ATP-Induced mTOR Activation in Reaction to 5-FU

**Figure 2:**
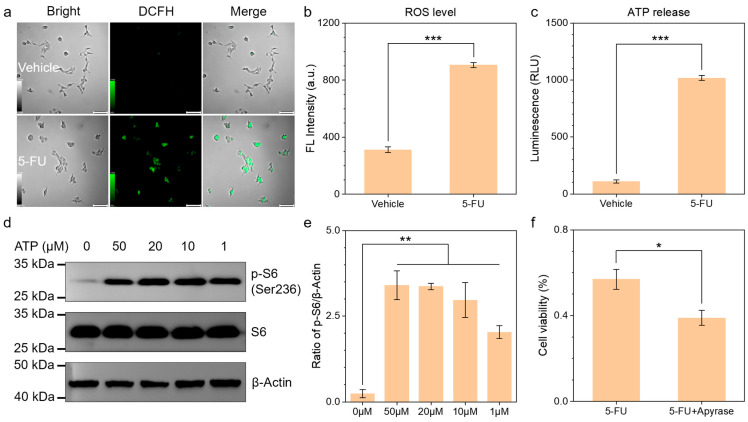
(**a**,**b**) DCFH fluorescence diagram (**a**) and quantitative analysis (**b**) of 4T1 cells with or without 5-FU treatment (5-FU, 5 μM) (scale bar, 100 μm). (**c**) ATP produced outside cells with or without 5-FU treatment (5-FU, 5 μM). (**d**,**e**) Western blotting analysis (**d**) and quantitative analysis (**e**) following treatment with varying concentrations of ATP (1, 10, 20, and 50 μM) for 20 min. (**f**) Cell viability after 5-FU treatment with or without apyrase (5-FU, 5 μM; apyrase, 10 U mL^−1^). * *p* < 0.05, ** *p* < 0.01, *** *p* < 0.001.

The therapeutic effect of 5-FU on tumors can be reversed by NAC, suggesting that intracellular reactive oxygen species (ROS) generation is essential for its anticancer activity. The enhanced 2′,7′-Dichlorodihydrofluorescein (DCFH) fluorescence observed in 5-FU-treated cells corroborates this hypothesis (Figure 2a). The quantitative analysis of fluorescence values showed a significant statistical difference between the two groups (Figure 2b). mTOR is a key protein involved in the resistance of apoptosis to ROS production, and CQ may enhance the cytotoxic effect of 5-FU by inhibiting the amino acid-induced activation of mTOR. To investigate this, we first validated the activation of mTOR in 4T1 cells. The 5-FU treatment group released a substantial amount of ATP into the extracellular environment due to cell death (Figure 2c). ATP within a concentration range of 50 μM to 1 μM was found to activate mTOR, leading to the phosphorylation of its downstream ribosomal protein S6 (Figure 2d,e). Consequently, the addition of apyrase to deplete extracellular ATP during 5-FU treatment significantly enhanced the therapeutic efficacy of 5-FU (Figure 2f). These results suggest that ATP-triggered mTOR activation contributes to the resistance of tumors to 5-FU treatment.

### 3.3. CQ Inhibits ATP-P2X4-mTOR Pathway

**Figure 3:**
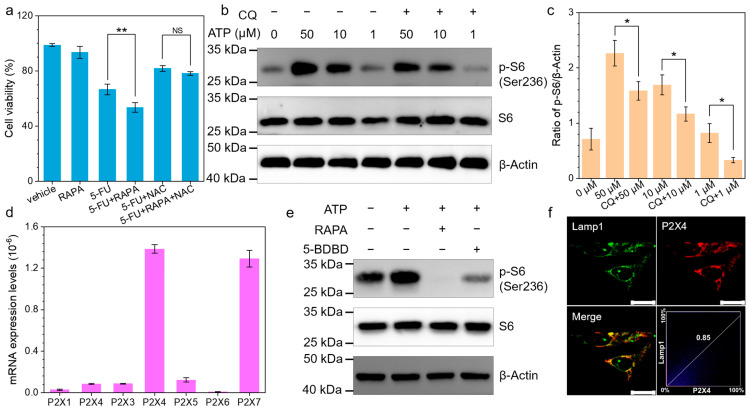
(**a**) Cell viability of different treatment groups (5-FU, 5 μM; CQ, 25 μM; NAC, 2 mM). (**b**) Immunoblots of 4T1 cells after incubation with different concentrations of ATP. CQ was added 2 h before treatment with ATP. (**c**) β-Actin was used as a loading control and the expressions of p-S6 were quantified. (**d**) RT-qPCR was used to analyze the expression of P2X family in 4T1 cells. (**e**) Immunoblots of 4T1 cells treated as indicated for 20 min. Inhibitors were added 20 min before treatment with 50 µM ATP. (**f**) Fluorescence microscopy images and colocalization analysis of P2X4 and Lamp1 immunostaining (scale bar, 20 μm). NS *p* > 0.05, * *p* < 0.05, ** *p* < 0.01.

The depletion of extracellular ATP significantly enhances the cytotoxicity of 5-FU. Subsequently, we employed rapamycin (RAPA) (mTOR inhibitor) to markedly increase the toxicity of 5-FU, and, additionally, NAC was able to negate the synergistic effect of RAPA treatment (Figure 3a). This suggests that the mTOR protein may play a key role in the synergistic therapeutic effect of CQ with 5-FU. The Western blot analysis confirmed that CQ significantly inhibits the phosphorylation of the mTOR downstream protein ribosomal protein S6 at concentrations ranging from 50 μM to 1 μM (Figure 3b,c). To elucidate this phenomenon, we considered the ATP receptor P2X4 located in the lysosome. Schmitt et al. has demonstrated that P2X4 can transmit extracellular ATP signals to activate mTOR in colorectal cancer [23]. Similarly, in 4T1 cells, P2X4 exhibited the highest expression within the P2X family (Figure 3d). The administration of RAPA and 5-BDBD (P2X4 inhibitor) significantly suppressed ATP-induced mTOR activation (Figure 3e), confirming the presence of the ATP-P2X4-mTOR pathway in 4T1 cells. Furthermore, consistently with reports in the literature, P2X4 preferentially localizes within the lysosome (Figure 3f).

### 3.4. CQ Promotes 5-FU Through P2X4-mTOR Inhibition

**Figure 4:**
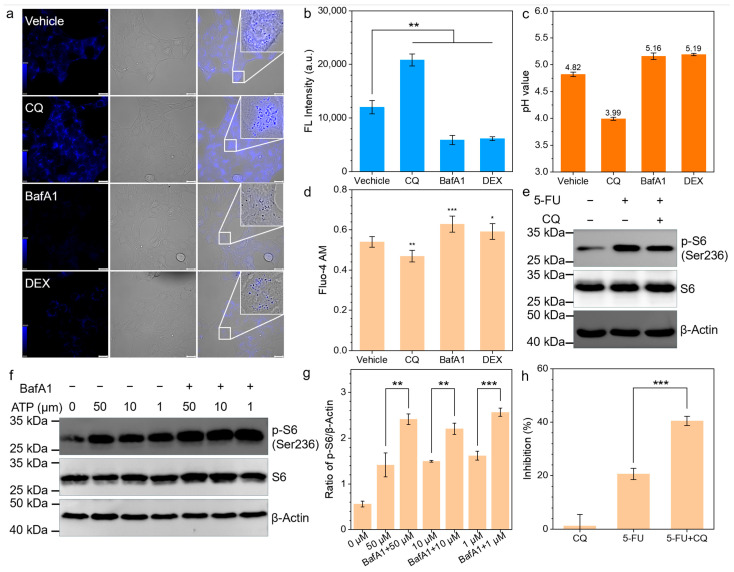
(**a**,**b**) Fluorescence images (**a**) and quantitative analysis (**b**) of using lysosomal acid sensitive probe to detect lysosomal pH value under different treatments (CQ, 25 μM; BafA1, 1 μM; DEX, 100 μM) (scale bar, 100 μm). (**c**) Lysosomal pH value detected by LysoSensor PDMPO after different treatments. (**d**) Intracellular calcium levels were quantified using Fluo-4 AM under different treatments, with ATP administration 20 min preceding the measurement. (**e**) Immunoblots of 4T1 cells treated as indicated. CQ was added 2 h before treatment with 5-FU. (**f**) Immunoblots of 4T1 cells after incubation with different concentrations of ATP. BafA1 was administered 2 h prior to ATP exposure. (**g**) β-Actin was used as a loading control and the expressions of p-S6 were quantified. (**h**) Quantification of 4T1 cell growth inhibition under different treatments. * *p* < 0.05, ** *p* < 0.01, *** *p* < 0.001.

CQ can alkalinize lysosomal pH, which may affect the activity of lysosomal P2X4 through this mechanism [24]. We attempted to verify the effects on lysosomal pH using CQ, bafilomycin A1, and dexamethasone (DEX). Interestingly, we found that in tumors, the impact of CQ on lysosomal pH was opposite to that of the other two lysosomal alkalinizing agents. An acid-sensitive lysosomal probe exhibited the strongest fluorescence signal in CQ-treated cells, indicating that the lysosomal pH was lower than in the other treatment and vehicle control groups (Figure 4a,b). We further assessed lysosomal pH changes using a ratio lysosomal probe (Figure 4c), calculating the lysosomal pH value following CQ treatment as 3.99 through a standard curve (Supplementary Figure S2), which was significantly lower than that of the other control groups. The activation of P2X4 triggers Ca^2^⁺ release; thus, we measured Ca^2^⁺ release induced by the ATP activation of P2X4 using Fluo-4 AM to indirectly assess P2X4 activity. The Fluo-4 AM fluorescence intensity in the CQ-treated group was the lowest, indicating that P2X4 activity was inhibited by CQ (Figure 4d). In contrast, the treatment of cells with bafilomycin A1, which increases lysosomal pH value, resulted in a significant increase in the phosphorylation of ribosomal protein S6 (Figure 4f,g). During the 5-FU treatment of tumors, the phosphorylation level of ribosomal protein S6 in the CQ-treated group was significantly lower than that in the untreated group (Figure 4e). Similarly, the MTT assays confirmed that CQ significantly enhanced the inhibitory effect of 5-FU on tumor proliferation. These findings suggest that CQ can inhibit mTOR activation through the ATP-P2X4-mTOR pathway, thereby increasing the chemosensitivity of 4T1 cells.

### 3.5. In Vivo Validation of CQ-Mediated Chemosensization

**Figure 5:**
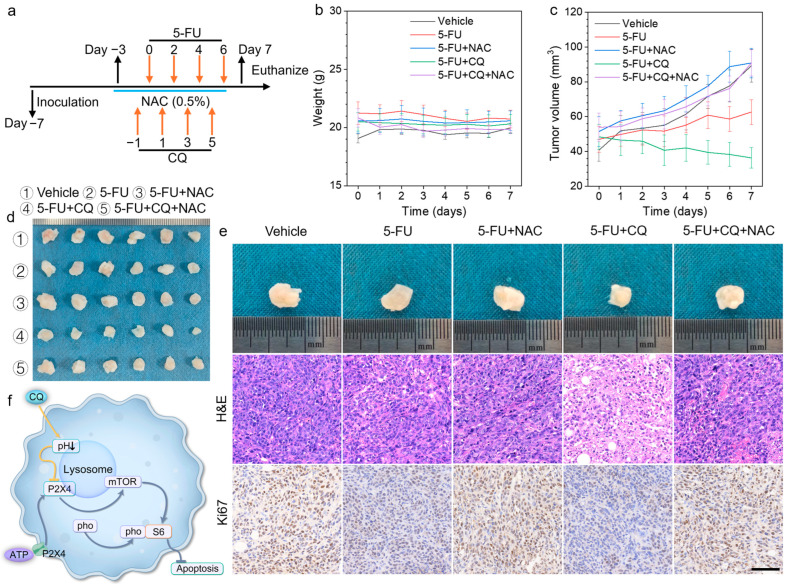
(**a**) Treatment region of 4T1 tumor-bearing mice (5-FU, 20 mg kg^−1^; CQ, 50 mg kg^−1^; NAC, 0.5%). (**b**) Body weight changes in different treated groups (*n* = 6). (**c**) Tumor volume (mm^3^) of different groups (*n* = 6). (**d**) Photograph of tumors dissected from each group on 7th day. (**e**) Representative images of subcutaneous 4T1 tumors and hematoxylin and eosin (H&E) and Ki-67 staining (scale bars, 100 µm) of tumor tissues of mice treated with 5-FU ± CQ as indicated. (**f**) Mechanistic schema of chloroquine-induced mTOR signaling blockade via lysosomal pH modulation in 4T1 cells.

The results demonstrate that chloroquine (CQ) synergistically enhanced the antitumor efficacy of 5-FU by augmenting the cytotoxic potency of 5-FU-generated ROS, ultimately inducing tumor cell apoptosis. To evaluate the in vivo antitumor efficacy, we treated BALB/c mice transplanted with 4T1 xenografts on different conditions (Figure 5a). During the treatment period, there were no significant differences in body weight among the groups, suggesting that the treatment resulted in no significant systemic toxicity in the mice (Figure 5b). Notably, Figure 5c illustrates that mice treated with the vehicle showed the fastest tumor growth, while the 5-FU + CQ group exhibited the most significant antitumor effect. Next, tumor tissues were excised and photographed (Figure 5d) to visually demonstrate the antitumor activity. As expected, the 5-FU + CQ group displayed the smallest tumor volume (Figure 5d), underscoring the considerable enhancement of antitumor activity in BALB/c mice transplanted with 4T1 xenografts when treated with 5-FU + CQ. At the endpoint, tumor sections were stained with H&E and Ki67 immunohistochemistry (Figure 5e). The results from H&E and Ki67 staining indicate that CQ significantly enhances the 5-FU effect, resulting in more efficient tumor inhibition.

## 4. Discussion

CQ, a traditional antimalarial medication [25], is currently being tested in clinical trials for its potential use in cancer treatment [26]. Its anticancer effects are thought to stem from reducing autophagy in tumor cells and aiding in the normalization of tumor blood vessels by targeting endothelial cells [27,28]. Notably, our study reveals a novel mechanism in 4T1 breast cancer models: CQ enhances chemosensitivity to 5-fluorouracil (5-FU) by suppressing the ATP-mTOR signaling pathway. This discovery provides new molecular insights to support its clinical translation as an adjuvant chemotherapeutic agent.

Consistently with previous studies [21,29], we confirm that CQ can enhance the antitumor effects of 5-FU. The EDU proliferation assay revealed that the chemosensitizing effect of CQ on 5-FU exhibited marked ROS dependency, as evidenced by N-acetylcysteine (NAC) abolishing this enhancement through free radical scavenging [22]. This confirms CQ’s role in amplifying 5-FU-induced oxidative stress. Further investigation uncovered that extracellular ATP-mediated mTOR activation—a well-established mechanism of chemoresistance—in 4T1 cells was suppressed by CQ through the disruption of lysosomal P2X4 receptor functionality (Figure 5f). Notably, we observed that CQ induced the acidification of the tumor lysosome, which contrasts with previous studies [30,31]. Mechanistically, this pH shift appears attributable to hyperactivation of the V-ATPase complex [32,33], which diminishes P2X4 receptor activity within acidified lysosomes [34,35], thereby blocking ATP-triggered mTOR signaling transduction. Finally, the regulatory effect of CQ on the ATP-P2X4-mTOR signaling axis and its chemosensitizing potential were further validated using a tumor-bearing murine model.

The murine model employed in this study demonstrated a high conservation of core mTOR signaling regulatory mechanisms with human systems. Critical pathway components—including insulin-like growth factor 1-mediated mTOR activation patterns [36] and lysosomal P2X4 receptor functional localization [35]—exhibit cross-species conservation. Furthermore, Wu et al. successfully modeled in murine systems the mechanism whereby CQ modulates feeding behavior via hypothalamic mTOR signaling suppression, demonstrating functional congruence with the mTOR-mediated ROS sensitivity regulation elucidated in our current investigation [37]. However, proteomic profiling revealed species-specific metabolic divergence: murine tumors predominantly rely on fatty acid degradation and FOXO pathway activation for energy homeostasis, whereas human neoplastic cells preferentially utilize galactose metabolism coupled with JAK-STAT signaling [36]. This metabolic rewiring underscores that while murine models effectively recapitulate central mTOR signaling dynamics, caution should be exercised when extrapolating metabolic toxicity profiles to clinical settings.

Of particular significance is the potential crosstalk between the CQ-mTOR regulatory axis identified in this study and AMPK signaling. Previous work has established that CQ induces AMPK activation through lysosomal stress [8], while the combinatorial use of AMPK activators with mTOR inhibitors has demonstrated synergistic tumor growth suppression with reduced mTOR inhibitor dosage [9]. Our findings that CQ exerts mTOR axis inhibition provide mechanistic corroboration for the functional interplay between AMPK/mTOR signaling and CQ’s antitumor efficacy. These insights not only advance our understanding of CQ’s molecular pharmacology in oncology but also lay the groundwork for developing dual-targeting strategies against both AMPK and mTOR pathways. Specifically, the lysosomal acidification-mediated mTOR inhibition observed here, when combined with AMPK activation, could establish a self-reinforcing therapeutic loop that amplifies metabolic disruption in malignant cells. Furthermore, the dose-sparing effects of such combination regimens hold particular promise for overcoming the dose-limiting toxicities associated with conventional mTOR inhibitors [9], warranting preclinical validation in patient-derived xenograft models and subsequent phase Ib dose-escalation trials.

In summary, the data in this study clearly demonstrate that CQ enhances the therapeutic efficacy of 5-FU by inhibiting ATP-induced mTOR activation. These findings may reveal the true mechanisms by which CQ aids antitumor effects and explore alternative approaches for mTOR blockade.

## Data Availability

The original contributions presented in this study are included in the article/Supplementary Material. Further inquiries can be directed to the corresponding authors.

## Supplementary Materials

The following supporting information can be downloaded at: https://www.mdpi.com/article/10.3390/biomedicines13040948/s1, Figure S1: Cell viability of 4T1 in different concentration of CQ; Figure S2: Standard curve of lysosome pH value.

## Abbreviations

AMPK	AMP-activated protein kinase
ATP	Adenosine triphosphate
BafA1	Bafilomycin A1
CHKA	choline kinase alpha
CQ	Chloroquine
DEX	Dexamethasone
LAMP1	Lysosome-associated membrane protein 1
mTOR	Mammalian target of rapamycin
NAC	N-Acetylcysteine
PFKM	Phosphofructokinase muscle
p-S6	Phosphorylated ribosomal protein S6
ROS	Reactive oxygen species
RAPA	Rapamycin
S6	Ribosomal protein S6
5-FU	5-Fluorouracil
DCFH-DA	2′,7′-Dichlorodihydrofluorescein diacetate
DCFH	2′,7′-Dichlorodihydrofluorescein
