# Supplementary material for: Chloroquine Enhances Chemosensitivity of Breast Cancer via mTOR Inhibition
Source: Biomedicines. 2025 Apr 12;13(4):948. doi: 10.3390/biomedicines13040948 (PMC12024896; doi:10.3390/biomedicines13040948)
Supplement: Supplementary file 1 [file biomedicines-13-00948-s001.zip › biomedicines-3508154-supplementary.pdf]

Supporting Information

Chloroquine Enhances Chemosensitivity of Breast Cancer via mTOR Inhibition

Zhihao Lin [1,†], Yuting Xu [1,†], Mifang Li [2], Yibiao Liu [2], Jianbo Yu [2,*] and Lingyan Zhang [1,2,3,4,*]

1. Shenzhen Clinical Medical College, Guangzhou University of Chinese Medicine, Shenzhen 518116, China; 20221111591@stu.gzucm.edu.cn (Z.L.); 20221111594@stu.gzucm.edu.cn (Y.X.)
2. Longgang Central Hospital of Shenzhen, Shenzhen 518116, China; 15626430473@163.com (M.L.); liuyibiao12345@126.com (Y.L.)
3. Lab of Molecular Imaging and Medical Intelligence, Department of Radiology, Longgang Central Hospital of Shenzhen, Shenzhen 518116, China
4. Longgang Clinical Institute of Shantou University Medical College, Shenzhen 518116, China

* Correspondence: jianbo___yu@126.com (J.Y.); 18819818005@163.com (L.Z.); Tel.: +86-135-3769-5609 (J.Y.); +86-188-1981-8005 (L.Z.)

† These authors contributed equally to this work.

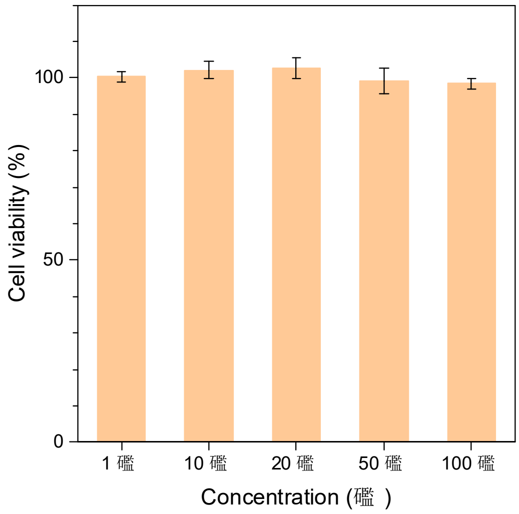

Supplementary Figure S1. Cell viability of 4T1 in different concentration of CQ.

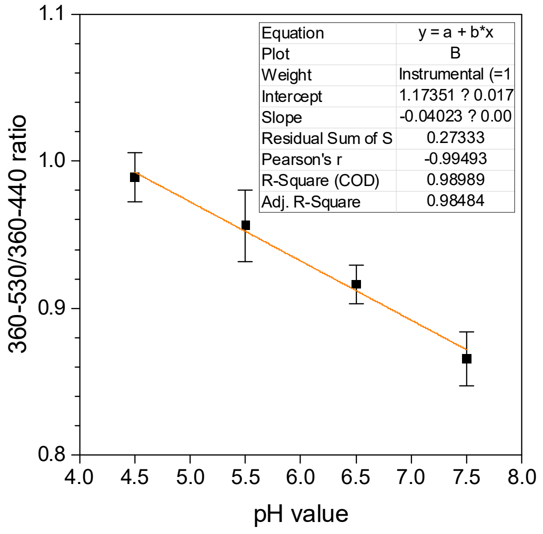

Supplementary Figure S2. Standard curve of lysosome pH value.
